# The Properties and Utility of Less Evaluative Personality Scales: Reduction of Social Desirability; Increase of Construct and Discriminant Validity

**DOI:** 10.3389/fpsyg.2020.560271

**Published:** 2020-10-27

**Authors:** Martin Bäckström, Fredrik Björklund

**Affiliations:** Department of Psychology, Lund University, Lund, Sweden

**Keywords:** big five, personality measurement, personality structure, self-ratings, social desirability

## Abstract

Evaluative neutralization implies rephrasing items such that it is less clear to the respondent what would be a desirable response in the given population. The current research compares evaluatively neutralized scales measuring the FFM model with standard counterparts. Study 1 reveals that evaluatively neutralized scales are less influenced by social desirability. Study 2 estimates higher-order factor models for neutralized vs. standard five-factor scales. In contrast to standard inventories, there was little support for higher-order factors for neutralized scales. Study 3 demonstrates the convergent and discriminant validity for the neutralized scales, e.g., by less inflated correlations to external measures. It is argued that evaluatively neutralized inventories help researchers come to grips with social desirability in personality measurement, and are particularly useful when the factor structure is central to the research question and there is a focus on discriminant validity.

## Introduction

Five factor inventories are used frequently in the behavioral sciences (Baumeister et al., 2007). They are also common in recruitment and assessment across the globe (Steiner, 2012). Unfortunately, however, many of the inventories reveal rather high correlations between the factors (Block, 1995). This is problematic since it violates a basic assumption of the underlying five-factor model (FFM); that is, that the five dimensions are separate or independent (Hofstee, 2003). Although some interpret the intercorrelation between factors in FFM-inventories as supporting a General Factor of Personality (GFP; Musek, 2007; Rushton et al., 2008), others see it as a nuisance factor and have made attempts to reduce it.

Research indicates that the correlation between factors is largely related to social desirability (e.g., Anusic et al., 2009; Bäckström et al., 2009), and the main strategy for achieving a purer measure of personality has been to reduce the influence of socially desirable responding (Paulhus and Vazire, 2007). One way to do this is to accept that personality inventories are influenced by social desirability and make adjustments after the data have been collected, e.g., by controlling for it statistically using separate measures. Another way is to employ an ipsative response format, where the rater is forced to choose between items that are comparable in desirability. Both of these methods have been criticized (e.g., Paulhus and Vazire, 2007), not least since they remove variance related to personality, which decreases the inventory’s construct validity and possibly also its criterion validity (McCrae and Costa, 1983). Recent advances in ipsative assessments, such as the multidimensional forced choice approaches (Brown and Maydeu-Olivares, 2013; Lee et al., 2019) have overcome some of the previous limitations and hold promise for the future. Although reliability-related questions still remain regarding the measurement of personality (see e.g., Seybert and Becker, 2019) which in turn may have consequences for validity, ipsative assessments may be a viable alternative for reducing socially desirable responding in some contexts.

Another strategy has been to create inventories through selection of items that show little sign of socially desirable responding (e.g., Jackson, 1984). With a few exceptions, however, these inventories do not concern the five-factor model, and many of them are difficult to come by or are expensive to use because they are owned by companies. The current research is an attempt to increase the understanding of why and how evaluativeness in five-factor model scales leads to flawed personality-measurement, and at the same time remedy the lack of evaluatively neutralized scales.

### The Current Study: Investigating the Desirability and Validity of Evaluatively Neutralized Big Five Scales

The scales used in the present study are new but the starting point was the inventory in the International Personality Item Pool (IPIP; Goldberg et al., 2006) that mimics the Revised NEO Personality Inventory (NEO-PI-R). Just like many personality inventories it contains evaluative items, and to reduce the evaluativeness of the items the evaluative neutralization method (Bäckström and Björklund, 2013) was adopted. Evaluative neutralization involves rephrasing items so that it is less clear to the respondent whether rating high or low is the more socially desirable response. The positive ring of an item such as “Like order” can be reduced by rephrasing it as the more neutral “Am only pleased if things are put in systematic order.” Similarly, the negative ring of an item such as “Avoid contact with others” can be made more neutral by rephrasing it as “Feel at ease even with being alone.” The evaluativeness of an item is important since it is related to item popularity (i.e., the item’s mean rating in the population) which, in turn, is an indicator of social desirability (Wahler, 1965). Items are more strongly related to desirability if the mean responses to them deviate from the midpoint of the response scale (Bäckström and Björklund, 2013). Items with high mean ratings (after reversing negative items) are more popular in the population, perhaps because they refer to culturally or contextually normative content (Bou Malham and Saucier, 2016), and they tend to drive responses in the same direction since *some* people prefer to rate themselves desirably. This has the consequence that all measures with high mean ratings will be related to one another (the orthogonality problem, e.g., Saucier, 2002), not because of common personality content in the different traits but because the items elicit the motivation to respond in a socially desirable manner and that this motivation varies in the population. Two respondents who have essentially the same personality characteristics may provide different responses, to the extent that one of them is more influenced by feelings of how popular the given behavior is in the general population. Arguably, item popularity interacts with the motivation to socially desirable responding, which makes ratings multifactorial. Because there are stable individual differences in the motivation to socially desirable responding (Kwan et al., 2004), the correlation between personality inventories and other (evaluatively loaded) measures should be inflated. Research on the present kind of issue becomes more feasible when there is a well-functioning measure of the evaluative factor at hand. We will follow Bäckström and Björklund (2013) and operationalize the evaluative factor as the difference between the responses to an evaluative and a neutralized inventory. We investigate the relations between the evaluative factor, popularity of items, and the level of social desirability of items, using ratings of social desirability.

What are the consequences of evaluative content in five factor measures for the factor structure? Previous research has suggested that since respondents vary in how sensitive they are to the evaluativeness of personality items, this aspect of the items, rather than the descriptive personality-related content of them, gives rise to an evaluative factor (Bäckström and Björklund, 2013). More specifically, our model proposes that the evaluative factor emerges as a reaction to the evaluative content of items from all five factors, to create a general evaluative factor (Bäckström and Björklund, 2014). This conceptualization of a general evaluative factor is similar to the model of Anusic et al. (2009), who proposed that a large halo-bias factor resides above all the personality factors, but also two common content factors akin to Digman’s (1997) alpha and beta. Other models don’t emphasize the evaluative aspect, but rather propose overarching content factors. The most well-known are the GFP (Musek, 2007; Rushton et al., 2008; van der Linden et al., 2010), and models with two overarching content factors (Digman, 1997; DeYoung, 2006). We will compare these different kinds of models, as estimated with evaluative vs. less evaluative scales (that have been subjected to evaluative neutralization). Which model has the best fit to the data, and is model fit dependent on how evaluatively loaded the items are?

The inclusion of evaluative personality items in personality scales should bring about inflated correlations to evaluatively loaded criteria, including many of those that are currently being investigated in psychology, e.g., psychological health, subjective well-being, and the like. The reason for this is that respondents who have a high motivation for socially desirable responding will provide desirable responses not only to the big five items but the other items too. Recent articles on the relationship between personality and quality of life fuel this concern. There are correlations across the board between personality variables and quality of life measures, i.e., even for some personality variables where there is little theoretical reason to expect a relationship to quality of life. For example, as Ozer and Benet-Martínez (2006) conclude in their overview of relevant research, subjective well-being is strongly predicted by extraversion and neuroticism, but it is also moderately predicted by conscientiousness, agreeableness, and openness. Similarly, a recent meta-analysis (Anglim et al., 2020) found moderate to large correlations between all Big Five factors and well-being. Another relevant example is the relationship between the Big Five and core self-evaluation (Judge et al., 2002). Importantly, the pattern of correlations should change when using evaluatively neutralized scales. The purer personality measure provided by evaluatively neutralized scales should produce a clearer and more differentiated picture of the relationship between personality and criteria.

### Overview of the Studies

The present research replicates and extends previous studies on how the evaluative factor in personality inventories influences the structure and discriminant validity of those measures. We present three studies that each compares the benefits of scales that have been subjected to evaluative neutralization versus traditional personality scales. The comparison is important since it speaks to whether evaluatively neutralized scales may reduce the problems that individual differences in the motivation to socially desirable responding cause for personality measurement.

**FIGURE 1:**
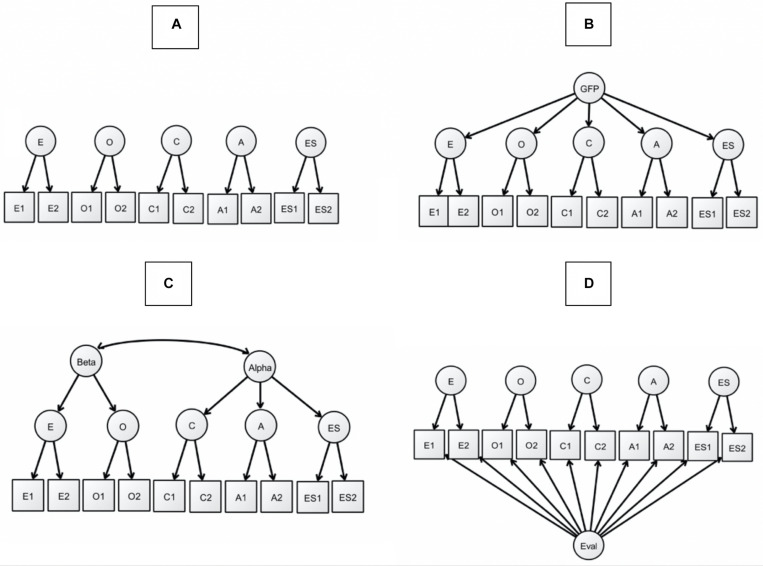
The four types of models tested in study 2. Panel **(A)** has the standard FFM model, panel **(B)** the GFP model, panel **(C)** the alpha beta model and panel **(D)** the bi-factorial evaluative model. The number of observed variables is lower than in the tested models.

The first study replicates the relation between the evaluative factor, the social desirability of personality items, and item popularity. It is expected that they will be closely related, but also that we will not find this relation in an inventory with more evaluatively neutral items. The second study replicates the finding that the evaluative factor can be attributed to popular items from all scales and factors of an FFM inventory. It also compares the evaluative factor model with other models that have been suggested to explain the correlation between factor scales of FFM inventories (see Figure 1, panel A-B). We estimate the evaluative factor model as a standard bifactorial model. In other words, a method factor related to all facets is added, to capture individual differences in evaluative responding. Such a model was delineated already by Edwards (1957), refined by Peabody (1967) and Saucier (1994), and was used in Bäckström and Björklund (2014). In addition to the evaluative factor model we also estimate a GFP-model (Musek, 2007; van der Linden et al., 2010) and an alpha beta model (Digman, 1997; DeYoung, 2006).

The third study investigates the influence of the evaluative factor on the correlation between personality and other psychological variables. When these measures are evaluative, there would appear to be a risk of inflated correlations, such that all five factors of an evaluatively loaded FFM-measure correlate significantly with evaluatively loaded external measures. Using evaluatively neutralized personality measures should provide a more diverse and theoretically reasonable pattern of correlations.

In all three studies we use two five-factor inventories: the Neutralized Big Five Inventory (NB5I) English and the NB5I Swedish. The inventories differ concerning the items that are used, but both are based on items from the IPIP-300 (Goldberg et al., 2006) that have been evaluatively neutralized, i.e., phrased in a way that makes them less susceptible to socially desirable responding, while retaining the capability of capturing personality content. Both inventories include the same subscales (facets), but are other otherwise independent (i.e., not translated variants). As opposed to many other inventories, they are free of charge and in the public domain.

## Study 1

In Study 1 we approach the question of whether evaluative neutralization produces items that are less loaded with social desirability. We investigate this by having a group of respondents rate the items’ level of social desirability, and then comparing the ratings of the evaluative and the neutralized items. We expect the neutralized items to be perceived as neither socially desirable nor undesirable. We also relate item social desirability with item popularity, defined as the extent to which the ratings deviate from the midpoint of the rating scale. We expect a positive correlation (as in Bäckström and Björklund, 2013, and Konstabel et al., 2006). Finally, we expect that both item social desirability and item popularity should be related to the general evaluative factor, which implies that socially desirable and popular items, respectively, should load stronger on the general evaluative factor.

### Materials and Methods

#### Participants

To rate the social desirability of the items 50 students, 26 women and 24 men, age *M* = 23.4, (*SD* = 5.8), were recruited at a Swedish university campus by a research assistant, who provided instructions and had them sign a consent form.

The evaluative factor was created by means of a Swedish Internet sample consisting 1167 participants, about 65% women. All scales were not rated by all subjects, but 367 participants responded to all included items. The respondents provided their responses on the www.pimahb.se website. Some of the participants had contributed in previous research studies at the same website before. Others responded just to get their personality profile after completing the ratings^1^

#### Measures

The participants provided their social desirability ratings on a website that was set up for this study. The total number of items was 546 (some from other inventories), but each participant only rated 300 items. Relevant for this study are the 120 items from the Swedish version of the Neutralized Big 5 inventory (NB5I) and 200 items from the IPIP-300 (items from the 20 sub-scales that are most similar the ones included in the NB5I, see Bäckström et al., 2014). The items were randomly distributed among the participants. The mean number of participants who rated an item was 28.9 (*SD* = 4.0). The response scale was a 9 -point Likert scale with labels beneath 1 (undesirable), 5 (neutral), and 9 (desirable).

To create the evaluative factor self-ratings on the 200 items from the IPIP-300 and of the 120 items from the Swedish version of NB5I were used. All items were rated using a 5-point Likert scale. The evaluative factor consisted of the difference between the factor scales of the evaluative IPIP-300 and the less evaluative NB5I (Swedish versions). This difference variable has been shown to correlate very highly (approximately 0.90) with a latent evaluative factor (Bäckström and Björklund, 2014).

### Results and Discussion

Were the evaluative items more socially desirable than the neutralized items? To test this, we compared the mean ratings of the Swedish IPIP-300 items with all neutralized items of the Swedish NB5I (negatively phrased items were reversed and all neuroticism items were reversed to measure emotional stability). The mean social desirability ratings were *M* = 6.55 (*SD* = 0.99) and *M* = 5.67 (*SD* = 0.74) for the evaluative and the neutralized items, respectively. This difference was highly significant, *t*(308) = 8.24, *p* < 0.001, *d* = 1.02^2^. This difference in ratings strongly supports that the neutralized items were in fact more neutral, compared to the IPIP-300 items. We also made pairwise comparisons of the subscales (facets) from the two inventories (see Table 1). Scale social desirability was defined as the mean item desirability of all items included in the sub-scale. Seventeen out of 20 scales of the IPIP-300 inventory had significantly higher social desirability ratings compared to the NB5I inventory. The exceptions were excitement seeking (neutral for both inventories), adventurous/variability seeking, and irritated. Generally, the items from scales/facets of the neuroticism factor of both inventories were rated very low in desirability. These results suggest that the scales of NB5I were more neutral compared to the scales of IPIP-300. As such, they replicate the findings from Bäckström et al. (2009).

**TABLE 1:** Means, standard deviations, and *p*-values for the social desirability ratings of the original IPIP-300 scales and neutralized versions.

	Original		Neutralized			
	*M*	*SD*	*M*	*SD*	*d*	diff *p*
Active	4.65	1.21	4.04	1.49	0.45	0.025
Friendly	6.21	1.08	4.38	1.25	1.57	<0.001
Happy	5.91	0.89	4.22	1.23	1.59	<0.001
Excitement seeking	4.02	0.99	3.83	1.36	0.16	0.376
Moral	5.63	1.15	4.83	0.95	0.76	0.001
Cooperative	5.28	0.87	3.79	1.17	1.46	<0.001
Altruistic	5.90	1.01	4.78	1.33	0.96	<0.001
Sympathetic	5.86	1.20	5.29	1.09	0.50	0.027
Self-efficacious	6.09	0.90	4.91	0.91	1.30	<0.001
Orderly	5.06	0.90	4.19	1.36	0.77	<0.001
Dutiful	6.36	1.10	5.16	1.13	1.08	<0.001
Achievement striving	5.72	0.86	4.08	1.03	1.74	<0.001
Anxious	2.58	1.51	2.95	1.62	0.24	0.051
Depressed	2.15	1.63	3.02	1.54	0.55	<0.001
Irritated	2.34	1.12	2.72	1.73	0.27	0.219
Vulnerable	2.31	1.33	2.71	1.44	0.29	0.011
Artistic	5.24	1.13	4.62	1.39	0.49	<0.001
Variability seeking	5.05	1.11	5.00	1.06	0.05	0.833
Intellectual	5.49	1.27	4.48	1.47	0.74	<0.001
Inner life	5.06	0.96	4.61	1.02	0.45	0.049

To estimate whether social desirability ratings were related to the evaluative factor we conducted analyses on the item level as well as the scale level. All items, both the evaluative and the neutralized, were correlated with the evaluative scale (the difference between evaluative and neutralized factor scales). In a second step, all the 194 items’ correlations were correlated with all the items’ rated social desirability. For the evaluative items the correlation was *r* = 0.72, which suggests that the items’ correlation was stronger, and relatively more positive, to the evaluative factor when social desirability was high. The same correlation was only *r* = 0.09 for neutralized items from the NB5I. In addition, we found support for our operationalization of evaluativeness, i.e., using the mean rating of the items (referred to as popularity). The correlation between mean ratings and social desirability was *r* = 0.67 for the evaluative items (see Wiggins, 1968 for a discussion of this relation). Mean ratings correlated strongly with the evaluative factor, *r* = 0.68. These correlations were very strong, given that the alpha for the evaluative factor was estimated to be 0.58 (given this level of reliability disattenuated correlations to the evaluative factor approaches *r* = 1.0).

The models were tested by confirmatory factor analysis and using Maximum Likelihood estimation using the MPLUS program (Muthén and Muthén, 1998-2017). Estimations on the scale level corroborated the above results. This time the evaluative factor was estimated within a full five-factor SEM model based on the IPIP-300 factor scales (facets). The evaluative factor was constructed as a bi-factor with loadings on all of the factors scales (facets). The evaluative factor was defined as zero correlated (orthogonal) to the five factors. The mean loadings were about *M* = 0.44, but loadings also varied, *SD* = 0.22. The loadings from the evaluative factor were correlated with the scales’ mean social desirability ratings. The correlation between loadings and desirability was found to be *r* = 0.79. This strong correlation clearly suggests that scales rated high in desirability was also scales having strong relation to the evaluative factor.

We also used the sub-scales (facets) from the neutralized inventory to estimate a bi-factor resembling an evaluative factor. The same model as was used for the evaluative scales was tested, but this estimation did not converge. After restricting several parameters, we succeeded to estimate a bi-factor model, but the pattern of factor loadings did not resemble a general factor with positive loadings on all facets, instead some were positive and some negative. In addition, the variance of this factor was very small.

In summary, these results strongly support that the items’ level of social desirability in the original IPIP-300 results in an evaluative factor, but also that the social desirability of the items are strongly related to mean rating of the items (item popularity). Reversely, the neutralized inventory appears to have fewer evaluative items, and it was *not* possible to create a general factor similar to the factor extracted for the IPIP-300 inventory.

## Study 2

Study 1 suggested that some inventories have a higher number of desirable and popular items, and also that these items are the ones that the evaluative factor is most strongly related to. Study 2 extends this result and is an attempt to replicate previous research comparing evaluative and evaluatively neutralized inventories with regards to factor structure. This relates to the controversies in personality measurement concerning the factor structure of personality. In Study 2 we will investigate if evaluativeness can explain why there are one or more higher-order factors in many personality inventories. The study does not concern whether higher-order factors are important, whether they should be kept in inventories, or be dismissed as nuisance. Instead, the goal is to investigate if personality inventories may have higher-order factors because the items in the inventories are evaluative (Bäckström et al., 2009).

Four different predictions will be investigated. The first concerns whether there is a higher-order personality factor in the inventories (General factor of personality, GFP, Musek, 2007; Rushton et al., 2008). The second concerns whether alpha and beta contribute to the higher-order structure of the inventory given the GFP, i.e., whether there is one higher-order factor connected to Extraversion and Openness and another connected to the three other FFM-factors (Digman, 1997; DeYoung 2006). The third concerns whether there is an evaluative factor in the inventory (e.g., Anusic et al., 2009; Danay and Ziegler, 2011, Revelle and Wilt, 2013;, Bäckström and Björklund 2014). Fourth, we test whether the evaluative factor in the inventory approaches tau equivalence (i.e., whether all loadings to the general factor are the same), which would imply even stronger support for a common evaluative factor.

We use two different methods to test the predictions. The first simply compares the NB5I with well-known inventories that have a sizable evaluative factor. We expect that the evaluatively neutralized inventory will show little support of either GFP, alpha-beta, or an evaluative factor. The second makes use of item popularity (Wahler, 1965). We create new scales by sorting items on whether they are popular or not, and then go on to compare popular with less popular scales and investigate the consequence of popularity to scale correlations.

### Materials and Methods

#### Samples

The samples in Study 2 have different origins. Two are from Sweden and consist of Internet visitors to www.pimahb.se. One is the Eugene Springfield public domain sample with courtesy to Lewis Goldberg (Goldberg and Saucier, 2016) and included 857 subjects (501 for the IPIP-inventories), 478 female, mean age 50.8 (*SD* = 13.2). One sample is from the NEO-PI-R manual (Costa and McCrae, 1992), where it is described as a reasonably diverse normative sample with respect to ethnicity, education and age. It included 1000 subjects, half of them female (age ranging from 21-96). One sample consists of Prolific and MTurk responders (used for NB5I) and consisted of 661 subjects, 341 women, with a mean age of 25.11 years (*SD* = 3.59). One sub-sample (300 participants) is from the Prolific service and the other sub-sample (361 participants) is from the MTurk service (here a rather large number of careless responders were deleted as defined by very short response times, less than 2 s per item and/or repetitive responses, unfortunately we did not have control items and warning in this study). Most participants were university students in the age range of 18-30, but there was also a small number of older non-student participants. The Swedish sample used for NB5I consisted of 705 participants, 457 females, with a mean age of 24.6 (*SD* = 8.39). The sample was a mix of students (60%), mostly paid for their participation with a movie ticket, and visitors to the website pimahb.se, who participated out of interest in psychological instruments. A very large sample (used for Goldberg’s Factor Markers-100) consisted of Swedish visitors to the website pimahb.se, who participated out of interest in psychological instruments, *N* = 6044, 4067 females (178 did not report sex), mean age 29.8 (*SD* = 10.8).

#### Measures

*NB5I.* The study presents results mainly from NB5I English. The inventory has 120 items, with six items on each facet, the facets were not totally balanced on positive and negative items. The NB5I Swedish has the same number of questions and facets, and was balanced.

Concerning evaluativeness, the items of both inventories were in general neutral, as suggested by the mean ratings being close to the midpoint of the rating scale. The variability in the item ratings was comparable or better than for many similar inventories, probably because there were no floor or ceiling effects. The reliabilities of factors were very high (close to 0.90), based on both Cronbach alpha and Omega estimations. Items generally loaded highly on their respective facets, even if there is room for improvement regarding this criterion. The five-factor structure was clear, and the inventories had few large secondary loadings between facets and factors (none higher than 0.30). The factor structure did not perfectly fit to an orthogonal five factor model, but some fit indices suggested an improvement in comparison to other inventories (e.g., the IPIP-300 or the NEO-PI-R). Both inventories had a loading structure that was invariant in relation to sex but showed expected differences in factor means and some facet intercepts. The correlation between the NB5I factors and the corresponding factors in the other big five inventories were strong (see also Bäckström et al., 2014, where a predecessor of the instrument was used, and Study 3).

*NEO-PI-R.* NEO-PI-R is one of the most frequently used inventories to measure the Big 5 (Costa and McCrae, 1992). It consists of 240 items distributed on five factors with six facets to each factor. Facet correlations, mean values and standard deviations were taken from the manual (Costa and McCrae, 1992).

*BFI-2.* The Big Five Inventory-2 (Soto and John, 2017) is the new version of the BFI which has been a popular short inventory to measure the Big five factors. It consists of 60 items distributed on 15 facets, with four items per facet. All facets are balanced in relation to positively and negatively phrased items. In Soto and John’s (2017) psychometric evaluation, the factor scales and the facets have good reliability and validity. Cronbach’s alphas of the facets in the present sample was very good, with all above 0.73 and some as high as 0.85. Cronbach’s alphas for the factors were 0.88, 0.87, 0.91, 0.92, and 0.89 for Extraversion, Agreeableness, Conscientiousness, Emotional stability (Negative Emotionality) and Openness (Open-Mindedness), respectively. Throughout the text we will only use the Big Five names for the all factors.

*GFM-100.* Goldberg’s (1992) Factor Markers (GFM-100) is an inventory from the IPIP set of Big-five inventories. It consists of 100 items, 20 items per factor. In the models, we have summarized the items in three random parcels, 3-4 items in each^3^. To maximize the evaluativeness of the inventory, a subset of the items was selected, based on item popularity. The 50 items with the highest mean ratings (after reversal of negative items) were selected to the GFM-100 Most Eval. In two samples, we contrasted this scale with inventories based on the 50 least evaluative/popular items, GFM-100 Least Eval. The English versions of the GFM-100 inventories were tested using the Eugene-Springfield sample, while the Swedish versions were tested and compared using the large Internet sample from pimahb.se. The inventory is the same as the one in Bäckström et al. (2009). The Cronbach’s alphas for all factors and all inventories were above 0.70.

Four different models will be tested on all inventories, the FFM model, the GFP model, the alpha-beta model and two versions of the evaluative factor model (see Figure 1, panel A–D). The last model is based on a bi-factorial model where the evaluative factor is extracted from the common variance of all observed variables. In one version loadings are free and in the other they are fixed to 1 (testing tau-equivalence). The four models are shown in Figure 1. The models will be tested by confirmatory factor analysis and using Maximum Likelihood estimation using the MPLUS program (Muthén and Muthén, 1998-2017). The research question concerns whether the GFP, alpha-beta, and the evaluative factor model substantially contribute to the personality factor structure. A substantive contribution of a delta Comparative Fit Index (CFI) larger than 0.02 will be used as cut-off value, implying that the more complex model have reduced the discrepancy between model and data by more than 2%, with some compensation for parsimoniousness. In any case, all changes of CFI larger than 0.01 were highly significant (*p* < 0.01) in all samples.

### Results and Discussion

All models tested are presented in Table 2. The first model estimated for all inventories was the FFM model with zero correlation between facets. It is obvious that for none of the inventories there was good fit. The best inventories were the NB5I and the GFM-100 (especially the least evaluative ones), but some of the other inventories did not fit very well to this model^4^. Of course, this makes it more difficult to test models since many inventories seem to have a more complex structure than the FFM.

**TABLE 2:** Fit indices of the FFM model, the GFP model, the alpha and beta model, the evaluative factor model and the tau equivalent evaluative model estimated from included inventories.

		Model									
		FFM		GFP		alpha beta		Eval factor		Eval factor tau	
Inventory	*N*	CFI	RMSEA	CFI	RMSEA	CFI	RMSEA	CFI	RMSEA	CFI	RMSEA
NB5I English	737	0.862	0.086	0.875	0.084	0.875	0.083	0.910	0.074	0.869	0.084
NB5I Swedish	705	0.844	0.089	0.850	0.087	0.855*	0.087*	0.880	0.082	0.847	0.088
IPIP-300 Swedish (20 facets)	1266	0.564	0.181	0.682	0.157	0.682	0.158	0.759	0.142	0.612	0.165
BFI-2 English	249	0.722	0.165	0.835	0.130	0.839*	0.128*	0.907	0.104	0.841	0.125
GFM-100 Least Eval	6044	0.852	0.111	0.914	0.087	0.884*	0.116*	0.914	0.103	0.912	0.086
GFM-100 Most Eval	6044	0.817	0.133	0.942	0.077	0.942	0.077	0.953	0.073	0.924	0.086
GFM-100 Least Eval ES	501	0.888	0.085	0.921	0.073	0.923	0.073	0.953	0.060	0.897	0.082
GFM-100 Most Eval ES	501	0.829	0.114	0.925	0.078	0.926	0.078	0.944	0.071	0.906	0.085
NEO-PI-R Manual	1000	0.581	0.114	0.629	0.108	0.639	0.107	x	x	0.618	0.109
NEO-PI-R ES	857	0.561	0.126	0.592	0.122	0.597	0.121	x	x	0.584	0.123
NEO-PI-R Most Eval scales Manual	1000	0.566	0.143	0.665	0.127	0.679	0.124	x	x	0.656	0.128
NEO-PI-R Most Eval scales ES	857	0.546	0.158	0.618	0.147	0.645*	0.145	x	x	0.624	0.144

ES, Eugene Springfield sample. *Some estimation problems, e.g., negative variance. X, model did not converge, Eval, evaluative.

Table 2 displays the CFI and root mean square error of approximation (RMSEA) from confirmatory factor analyses of all included inventories. In relation to whether there was a general factor in the inventories, the columns under FFM and GFP can be compared (the latter model has 4 fewer degrees of freedom). The NB5I inventories did not show a substantive change in fit when GFP was added, but all other inventories showed substantially better fit. The most substantial changes were found for the IPIP-300, the BFI2 and the most evaluative variants of GFM-100. Also, the NEO-PI-R showed an increase when the GFP was added to the FFM model.

Generally, the estimations did not support the alpha-beta model (see Table 2). There were some estimation problems for some of the inventories, but for those that did not need more restrictions, only NEO-PI-R gave some support for the alpha-beta model (this model has 1 degree of freedom less than the GFP model), and this support was rather weak.

Two versions of a bi-variate model of the evaluative factor were estimated for the inventories. In the first version the loadings were free (in comparison with the FFM model, degrees of freedom were reduced with the corresponding number of observed variables) to vary and in the second they were restricted to be the same on all variables (this model has 1 degree of freedom less than the GFP model). Comparing the first and second variant tests something similar to tau-equivalence, i.e., that the loadings on all observed variables are the same. In both models the evaluative factor was defined as orthogonal to the FFM factors. Comparing the models with evaluative factors with the FFM model provides information about the strength of the evaluative factor, in other words how much of the covariation between the observed variables that can be attributed to an evaluative factor. One problem with the freely estimated bi-factor model is that there is no guarantee that the latent variable will consist of variability related to a general evaluative factor. A large discrepancy between the free model and the restricted tau model may suggest that there is no general factor or that some of the variables are not influenced by evaluative responding to the same degree. The last-mentioned suggestion is very plausible, given the variability in social desirability and popularity between scales of most inventories.

The model with an evaluative bi-factor was tested using the BF5I inventories. The models had a substantially better fit compared to the FFM model, delta CFI was 0.48 and 0.23, respectively. When testing for tau-equivalence, i.e., whether the factor is general, the CFI was only 0.007 higher compared with the FFM model for NB5I English, and only 0.004 higher for NB5I Swedish. The last analysis suggests that for neither inventory there was any evaluative factor summarizing variance from all facets of the inventory. The results suggest that there is some correlation between facets from different factors in the NB5I inventories, but it can neither be attributed to a GFP, alpha-beta nor an evaluative factor. The estimation of the evaluative bi-factor model revealed better fit for both the BFI-2 and the GFM-100. The tau model revealed somewhat worse fit, but much better than the FFM model. This suggests that there was a general factor, but that some of the variables loaded more strongly on this factor. Due to convergence problems, it was not possible to estimate the evaluative factor model for NEO-PI-R, but the fixed (tau equivalent) evaluative factor model converged. Generally, this model had better fit than the FFM model, and comparable or somewhat lower fit than the higher-order model. The fit of the NEO-PI-R data sets was comparatively worse than all other inventories, probably due to a large number of secondary loadings (alternatively sub-factors).

To summarize, the evaluative factor was not supported for the NB5I, but all other inventories had a rather strong higher order factor. This factor can be interpreted either as a GFP or as an evaluative factor, because these two models had an almost identical fit. As for the alpha beta there was almost no support for such a model in the estimations of the current set of inventories.

One explanation for why an evaluative factor appears in an instrument is that it contains popular items, i.e., items that are rated well above the midpoint (after reversal) of a typical Likert rating scale (Wahler, 1965). An inventory with a disproportionately large number of items or scales that have mean values above the midpoint should have a larger evaluative factor. To test this hypothesis, we used the two versions of the GFM-100 inventory, one based on the most evaluative and one based on the least evaluative items. The results are displayed in the lower part of Table 2 (GFM-100 Most Eval). The CFI of the FFM models were somewhat larger, but more importantly, the estimated CFI of the GFP and the evaluative model where larger. Another test of the same hypothesis was conducted based on NEO-PI-R. We selected the four facets of each factor that had the highest popularity, i.e., had the largest mean distance from the midpoint of the rating scale. Again, the fit of the FFM model was somewhat worse compared to the model which included all six facets (NEO-PI-R Most Eval), but the GFP model fitted relatively better, suggesting that there was more common correlation between scales from all factors. Also, the two evaluative factor models revealed better fit when the scales based on more popular items were used (e.g., the ones called eval in Table 2).

It appears that when more popular items or scales are included in an inventory the evaluative factor gets stronger. This supports the idea that popularity is a unique factor which influences ratings in a very general way (Bäckström et al., 2009; Bäckström and Björklund, 2016), an idea with roots that can be traced back at least to Peabody (1967) and problematizes the interpretation of a general factor in terms of personality substance. There has been support for interpreting the evaluative factor both as substance and as style. Anusic et al. (2009) identified what they called a halo factor and found no support for an interpretation of it in terms of substance, mainly since ratings from different observers of the same target were unrelated. Others have strongly suggested that the GFP/Evaluative factor can be given an interpretation in terms of substance (e.g., Rushton et al., 2008; van der Linden et al., 2010), more particularly social effectiveness (Dunkel et al., 2016; van der Linden et al., 2016). On a similar note, there was little support for a model with two overarching personality factors, i.e., models of the kind of the kind suggested by e.g., Digman (1997) and DeYoung (2006). Our results support Block’s (1995) contention that there is often rather high interfactor-correlation in personality inventories, and we propose that item popularity is an important reason for this.

An obvious limitation of these results is that some inventories do not fit the theoretical FFM model at all, suggesting that there was a large amount of covariance in the data not accounted for by the models. On the other hand, the FFM model is the most commonly used with these inventories, especially when scales are constructed based on summarizing items or sub-scales. Furthermore, the correlation between factors in the models were very similar to the correlations for scales, and this pattern of correlation is the one used for testing the GFP and the alpha-beta model. The evaluative factor, defined to be uncorrelated with the FFM factors, captures covariation between observed variables not accounted for by the other factors. The fact that almost all variables contribute to the evaluative factor in some inventories suggests that this factor is general and originates in something that all variables have in common.

A recent study (Bäckström and Björklund, 2016) approached the issue in an unorthodox way and showed that rephrasing the items of an evaluative five-factor measure to make them evaluatively neutral made the evaluative factor disappear, and rephrasing them again to make them more evaluative made the evaluative factor reappear. This suggests a clear link between item popularity and the evaluative factor. The influence of popular items on personality inventories was tested in the present work (Study 2), where it was found that selecting scales or items from NEO-PI-R and GFM-100, respectively, resulted in inventories with a stronger GFP/evaluative factor. Whether the evaluative factor is only a method factor or also has content, i.e., measures trait-like features, is still an open question.

## Study 3

Study 2 suggested that scales from inventories with a large number of evaluative/popular items generate an evaluative factor that is separate from the personality content factors. It appears that the evaluative factor can be related to any personality item that is normatively negatively or positively valued in the population (i.e., popular or not). Study 3 focuses on the validity issues that the evaluative factor may cause when the scales of the inventory are related to criteria, i.e., compared to measures of relevant concepts. One important issue is to clarify what the consequences are of using evaluative measures when the criteria are evaluative too. For example, when both the scales of the inventory and the variables used for validation are under the influence of evaluation, i.e., include both content and evaluation, they are multifactorial and there should be a risk for overestimation of the convergent validity. Furthermore, evaluative influence should weaken discriminant validity. Since evaluatively neutralized personality inventories have more independent factors (close to zero correlations, see Study 2 and Bäckström et al., 2009), using neutralized scales should entail higher discriminant validity. Similar lines of reasoning have been put forward by e.g., John and Robins (1993) and Leising et al. (2015). The present study will investigate the validity of neutralized and evaluative inventories with the expectation of finding comparable convergent validity but better discriminant validity for the neutralized scales. As such, it will replicate and extend some of the findings from earlier studies.

To investigate how evaluative vs. evaluatively neutralized inventories are related to criterion measures, the evaluatively neutralized measure (NB5I) will be compared with two other inventories measuring the Big 5, the BFI-2 (Soto and John, 2017) and the GMF-100 Eval. Convergent validity will be tested based on the correlation between the NB5I factor scales and the factor scales of BFI-2 and the GFM-100 Eval. High but not perfect correlations is expected, since at least the GFM-100 Eval is very evaluative, and Study 2 has suggested that also the BFI-2 is evaluative. The evaluative factor, being present in all scales of evaluative inventories, can cause an attenuated pattern of correlation between the neutralized and the more evaluatively loaded inventories, and controlling the correlations for this factor is expected to make the correlations either stronger or of similar magnitude. Moderate correlations (strong for the same factors) are also expected between most scales of the two evaluative inventories, but these will be reduced after controlling for the evaluative factor.

The test of criterion and discriminant validity will be based on core self-evaluation and life satisfaction measures, which were chosen since they concern aspects of personality that are desirable, and since they are measured using self-rating (global assessment) instruments that are common in the literature. Evaluative five factor scales will be compared to neutralized with respect to how the factors correlate with the Core self-evaluation measures (Judge et al., 2002, 2003) and a measure of life satisfaction (Diener et al., 1985). We expect fewer significant correlations to the core measures and life satisfaction measure when using evaluatively neutralized scales as compared to traditional measures. More precisely, we expect that the NB5I will show better discriminant validity, by having a more distinct pattern of correlation to core self-evaluation (expected correlation mainly to conscientiousness and emotional stability) and life satisfaction (expected correlation mainly to conscientiousness, emotional stability and extraversion).

We will also relate the evaluative factor to the core self-evaluative factor, expecting them to be correlated but distinct. Judge et al. (2002) proposed that estimates of neuroticism, self-esteem, generalized self-efficacy, and internal locus of control capture a single core self-evaluative factor. This factor has similarities with the evaluative factor, but is based on how respondents rate a particular factor of the Big 5, neuroticism, rather than all five. Respondents who have a negative view of themselves indeed often rate very high on the neuroticism factor, and since being anxious, worried, or depressed is considered negative in Western society people who perceive themselves this way will be regarded as not really liking themselves. Previous research has shown that the evaluative factor is related to but not the same as the neuroticism factor (Bäckström and Björklund, 2014). This can be taken to suggest that the part (variance) of the evaluative factor related to neuroticism is the same as that included in the core self-evaluative factor of Judge et al. (2002), whereas the other part is related to something specific to the evaluative factor (e.g., the extent to which one would like to align oneself with societal norms). This should have important consequences for how evaluative and evaluatively neutralized inventories relate to criterion measures. To the extent that the criteria are evaluative, correlations to evaluatively loaded (but not evaluatively neutralized) Big Five measures should be inflated.

### Materials and Methods

#### Participants

Two samples will be used in this study. One sample consists of 249 participants from the MTurk Service, 117 men and 132 women (mean age 24.9, *SD* = 3.31). Only those who were in the age range of 18-30 and involved in studying more than 50% were included. This sample is also part of the larger sample used in Study 2, but included the criterion measures. The other sample is a Swedish Internet sample consisting of 1037 men and 2013 women (mean age 23.18, *SD* = 3.75).

#### Materials

Both the NB5I English and NB5I Swedish (see Study 2) was used. In order to improve the balance between positive and negative items in the NB5I English, changes were made to three items in the activity facet, one in the moral facet, one in the non-anxious facet, and three negative items were exchanged for positive items in the inner-life facet. The resulting version had 120 items, with six on each facet, and all facets were balanced regarding negative and positive items. Reliability and factor structure was almost equivalent to those in Study 2. The factors, with and without the added and changed items, correlated 0.963, 0.977, 0.953, 0.959, and 0.953 for Extraversion, Agreeableness, Conscientiousness, Emotional stability and Openness, respectively. The NB5I Swedish had 160 items, with eight items on each facet. The reliability for factors was very high (close to 0.90 for all factors). The BFI-2 (Soto and John, 2017) that was used in Study 2 was used in Study 2 too.

The GFM-100 Eval used here was the same as GFM-100 Eval used in study 2 and includes the ten most popular items from each of the factors. The selection was made from the Eugene Springfield data set (Goldberg and Saucier, 2016). Selecting the most popular items should make the inventory very evaluative, and due to this, high correlations between the scales were expected. Cronbach’s alphas for the factors were 0.91, 0.89, 0.83, 0.90, and 0.85 for Extraversion, Agreeableness, Conscientiousness, Emotional stability and Openness, respectively.

The core self-evaluative factor was captured using measures of self-esteem, generalized self-efficacy and emotional stability. Self-esteem was measured using Rosenberg’s (1965) Self-Esteem scale (α = 0.92). Generalized self-efficacy was measured using Judge et al.’s (1998) scale (α = 0.92). In addition to this, core evaluations were measured with the Core Self-Evaluations scale (α = 0.94) from Judge et al. (2003). To measure life satisfaction, the Satisfaction with Life Scale (SWLS; Diener et al., 1985) was used. It concerns global life satisfaction and includes 5 items (α = 0.89).

### Procedure

For those who completed the English inventories, the materials were presented in the following order: The NB5I, the core self-evaluative factor scales together with Life satisfaction, the GFM-100 Eval (with only 50 items), the BFI-2. Items were presented to the participants without any pause, and the only thing indicating a change was that the heading changed from “Rate spontaneously” to “I am someone who.” when the BFI-2 items were introduced, in accordance with Soto and John (2017). For those who completed the Swedish inventories, items from different inventories were presented in random order.

### Results and Discussion

The first analyses concern the convergent and discriminatory validity of our proposed evaluatively neutralized measure, NB5I. To test the convergent validity, the factor scales were correlated with the factor scales of BFI-2 and GFM-100 Eval. Table 3 shows all correlations between the factor scales between NB5I and GFM-100 Eval (both language versions). There were rather high correlations to the corresponding factor scales of the NB5I and GFM-100 Eval. The factor with the lowest correlation was extraversion. The strongest correlation was found for the neuroticism factor, but there was also strong correlation between NB5I Emotional stability and all the other GFM-100 Eval scales, especially with Extraversion and Conscientiousness. One possible reason is that the GFM-100 Eval scales are evaluative and thereby correlated with the emotional stability factor of the NB5I, which shares content with the evaluative factor. The relations between the NB5I and the BFI-2 are displayed in the upper part of Table 4. Strong correlations were again found between the corresponding factor scales of the two inventories. The pattern with comparatively high correlation with emotional stability was found again. Again, the high correlations to corresponding factor scales support the convergent validity of the NB5I. Together with previous reports of convergent validity to NEO-PI-R and IPIP-NEO-PI (Bäckström et al., 2014) this suggests that neutralization does not decrease the convergent validity in a personality inventory.

**TABLE 3:** Convergent validity: Univariate correlations between the factor scales of NB5I English/Swedish and GFM-100 Eval, and partial correlations with control for evaluative factor.

		NB5I E	NB5I A	NB5I C	NB5I Es	NB5I O
English	GFM-100 E	0.55 (0.60)	0.00(−0.03)	0.20 (0.10)	0.48 (0.28)	0.10 (0.05)
	GFM-100 A	0.22 (0.20)	0.61 (0.67)	0.16 (0.08)	0.20(−0.05)	0.36 (0.32)
	GFM-100 C	−0.11(−0.18)	0.13 (0.12)	0.83 (0.85)	0.43 (0.23)	−0.04(−0.03)
	GFM-100 Es	0.09 (0.02)	0.16 (0.18)	0.27 (0.16)	0.88 (0.85)	0.09 (0.08)
	GFM-100 O	0.20 (0.17)	−0.04(−0.07)	0.18 (0.08)	0.27(−0.01)	0.60 (0.64)
Swedish	GFM-100E	0.69 (0.83)	−0.15(0.00)	0.17 (0.06)	0.41 (0.25)	0.18 (0.18)
	GFM-100A	0.22 (0.25)	0.56 (0.73)	0.15 (0.08)	0.18 (0.04)	0.27 (0.27)
	GFM-100C	−0.14(−0.15)	0.04 (0.18)	0.80 (0.80)	0.42 (0.16)	0.10 (0.09)
	GFM-100Es	−0.05(0.08)	−0.01(0.17)	0.35 (0.28)	0.90 (0.86)	0.07 (0.05)
	GFM-100O	0.11 (0.14)	−0.14(−0.01)	0.24 (0.15)	0.16(−0.05)	0.60 (0.65)

*Partial correlation controlling for evaluative factor in parenthesis.*

**TABLE 4:** Convergent validity: univariate correlations between the factor scales of NB5I English and BFI-2, and partial correlations with control for evaluative factor.

	NB5I E	NB5I A	NB5I C	NB5I Es	NB5I O
BFI-2 E	0.45 (0.47)	−0.02(−0.05)	0.30 (0.22)	0.50 (0.31)	0.12 (0.09)
BFI-2 A	0.14 (0.11)	0.67 (0.76)	0.22 (0.13)	0.40 (0.19)	0.12 (0.16)
BFI-2 C	−0.07(−0.14)	0.15 (0.15)	0.82 (0.86)	0.50 (0.31)	−0.05(0.05)
BFI-2 Es	0.14 (0.10)	0.07 (0.07)	0.33 (0.27)	0.88 (0.87)	−0.05(0.04)
BFI-2 O	0.17 (0.14)	0.07(−0.06)	0.05(−0.04)	0.22 (0.02)	0.77 (0.78)

*Partial correlation controlling for evaluative factor in parenthesis.*

Strong correlations indicating convergent validity were also expected between the two evaluative inventories, but also moderate correlations cross factors. Table 5 shows that this was exactly what was found.

**TABLE 5:** Convergent validity: univariate correlations between the factor scales of GFM English and BFI-2, and partial correlations with control for evaluative factor.

	BFI2 E	BFI2 A	BFI2 C	BFI2 Es	BFI2 O
GFM-100 E	0.88 (0.83)	0.28(−0.01)	0.31 (0.02)	0.60 (0.33)	0.23 (0.00)
GFM-100 A	0.24(−0.01)	0.79 (0.72)	0.28 (0.05)	0.24(−0.05)	0.40 (0.26)
GFM-100 C	31 (0.06)	0.32 (0.09)	0.88 (0.84)	0.44 (0.12)	0.07(−0.16)
GFM-100 Es	0.45(−0.13)	0.47 (0.17)	0.47 (0.15)	0.90 (0.80)	0.26(−0.04)
GFM-100 O	0.33 (0.07)	0.23(−0.06)	0.27 (0.02)	0.31(−0.15)	0.75 (0.69)

*Partial correlation controlling for evaluative factor in parenthesis.*

### Evaluative Factor

In Bäckström and Björklund (2014) it was shown that an evaluative factor could be extracted as a separate factor, parallel to the FFM-factors (rather than higher.-order). Can this factor explain the many strong correlations between scales of the BFI-2 and the GFM-100? To investigate this question, we used Confirmatory factor analysis using Maximum Likelihood estimation using the MPLUS program (Muthén and Muthén, 1998-2017) and first created a model with five latent variables, one for each factor of the big five. The observed variables were measures from the three FFM instruments used in this study. To estimate the common correlation between the factors, we added an evaluative latent variable based on all scales from the BFI-2 and the GFM-100 Eval. The evaluative factor has been shown to correlate with emotional stability (see Bäckström and Björklund, 2014), but in the analyses emotional stability was not included, so as to provide a stronger test of how much the correlations were weakened. The correlations between the FFM factors were fixed at zero and also the Evaluative factor was defined as uncorrelated to the FFM (just as in Bäckström and Björklund, 2014). The evaluative factor scores were extracted and thereafter used as a control variable in partial correlation between the NB5I and the other two measures. In Table 3-5 the partial correlations are shown in the parenthesis after the univariate correlation. Indeed, controlling for the evaluative factor made the correlation between variables originating from the same factors overall somewhat stronger for the NB5I, and the discriminant validity was more pronounced too, which is obvious in relation to the emotional stability factor (all partial correlations were lower, except to emotional stability). Even more dramatic changes were found for the correlations between the two evaluative inventories, convergent validity was still strong, but the cross-factor correlations were much weaker. To summarize, these correlations show strong support for both convergent and discriminant validity.

### Implications for Validity

The last part of the results concerns the criterion and discriminant validity of neutralized as compared to more evaluative inventories. Specifically, we investigated whether personality predicts scales from the core self-evaluation concept (both samples) and a scale measuring life satisfaction (only English sample). Table 6 (English) and Table 7 (Swedish) have both the univariate correlations and the partial correlations controlling for the evaluative factor. Generally, the less evaluative (neutralized) inventory showed high correlations between emotional stability and scales measuring core self-evaluation. There were also positive correlations with conscientiousness and in the Swedish sample a weak correlation to extraversion.

**TABLE 6:** Univariate and partial correlations between the factor scales of NB5I, BFI-2, and GFM-100 Eval with Core Self-Evaluations, Self-esteem, Self-efficacy and Life satisfaction in the English-speaking sample.

	Core self-evaluation	Self-esteem	Self-efficacy	Life satisfaction
NB5IE	0.11 (0.06)	0.10 (0.05)	0.12 (0.07)	0.27**(0.26**)
NB5IA	0.03 (0.01)	−0.01(−0.04)	−0.09(−0.15*)	0.11 (0.10)
NB5IC	0.47**(0.44**)	0.34**(0.27**)	0.41**(0.36**)	0.36**(0.29**)
NB5IEs	0.75**(0.64**)	0.66**(0.50**)	0.62**(0.45**)	0.63**(0.29**)
NB5IO	0.06 (0.06)	0.08 (0.05)	0.13*(0.13*)	0.13*(0.12)
BFI-2 E	0.56**(0.38**)	0.56**(0.34**)	0.46**(0.18**)	0.57**(0.38**)
BFI-2 A	0.38**(0.09)	0.34**(0.01)	0.29**(−0.06)	0.43**(0.20**)
BFI-2 C	0.62**(0.43**)	0.53**(0.29**)	0.57**(0.35**)	0.48**(0.26**)
BFI-2 Es	0.83**(0.69**)	0.76**(0.55**)	0.69**(0.43**)	0.72**(0.55**)
BFI-2 O	0.19**(−0.08)	0.23**(−0.04)	0.31**(0.07)	0.16**(−0.08)
GFM-100 eval E	0.55**(0.31**)	0.53**(0.27**)	0.50**(0.22**)	0.56**(0.36**)
GFM-100 eval A	0.24**(−0.07)	0.24**(−0.08)	0.22**(−0.11)	0.30**(0.05)
GFM-100 eval C	0.54**(0.34**)	0.43**(0.17**)	0.49**(0.26**)	0.42**(0.20**)
GFM-100 eval Es	0.69**(0.45**)	0.63**(0.32**)	0.61**(0.28**)	0.63**(0.39**)
GFM-100 eval O	0.34**(0.00)	0.36**(0.02)	0.51**(0.26**)	0.26**(−0.05)
Eval factor	0.63**	0.64**	0.64**	0.56**

Partial correlation controlling for evaluative factor in parenthesis. *p < 0.05, **p < 0.01.

**TABLE 7:** Correlation between the factor scales of NB5I and GFM eval with Self-efficacy and Self-esteem in the Swedish sample.

	Self-esteem	Self-efficacy
NB5IE	0.17**(0.21**)	0.12*(0.16**)
NB5IA	−0.18*(−0.05)	−0.16*(−0.03)
NB5IC	0.38**(0.32**)	0.48**(0.44**)
NB5IEs	0.76**(0.70**)	0.64**(0.55**)
NB5IO	0.02(−0.01)	0.15*(−0.15*)
GFM E	0.46**(0.27**)	42**(0.21**)
GFM A	0.20**(0.03)	0.19**(0.01)
GFM C	0.35**(0.17**)	0.45**(0.29**)
GFM Es	0.68**(0.56**)	0.55**(0.37**)
GFM O	0.27*(0.03)	0.49**(0.33**)

Partial correlation controlling for evaluative factor in parentheses. *p < 0.05, **p < 0.01.

A large part of the variability of the Core Self-evaluative factor was common with the evaluative factor extracted from the BFI-2 and the GFM-100 Eval (see last row of Table 6). After controlling for the evaluative factor, the partial correlations were more similar to the ones showed for the NB5I. After controlling for the evaluative factor, emotional stability, conscientiousness and to some extent extraversion contributed to Core Self-evaluation, but there were only very weak correlations to agreeableness and openness. These analyses also suggest that Core Self-evaluation scales includes unique content, distinct from what is included in the evaluative factor in this study.

The correlations were estimated in relation to the life satisfaction scale, in the English sample. The evaluative inventories showed positive correlation to all five factors, while the neutralized showed positive correlation mainly to emotional stability, conscientiousness and extraversion. When controlling for the evaluative factor the pattern of correlations for the neutralized and the evaluative inventories became much more similar.

Study 3 focused on the validity-related problems that the evaluative factor causes when the inventory is related to criterion measures. We predicted and found that when both the inventory and the variables used for validation are under the influence of evaluation the convergent validity is likely to be overestimated (see also Bäckström et al., 2014). Importantly, evaluatively neutralized inventories (e.g., NB5I) have higher discriminant validity in relation to the evaluative inventories (e.g., BFI-2 and the GFM-100) than these inventories have within their own factor scales. Our interpretation of this is that since neutralized inventories do not contain as much evaluative content, the relations between neutralized inventories and other (evaluative) inventories are to a higher degree based on personality content variability. The Emotional stability factor of neutralized inventories, however, is clearly more strongly related to all factors of evaluative inventories. This is also very interesting, since the evaluative factor is clearly related to Emotional stability in neutralized inventories (see also Bäckström et al., 2014, and similar findings in Borkenau and Ostendorf, 1989). It seems that the Emotional stability factor captures some of the variability of the evaluative factor. A possible interpretation of this is that many factor scales of other inventories are loaded by both evaluation and emotional stability. In any case, these results support the discriminant validity of neutralized inventories.

The results of Study 3 suggest that one reason why personality factors sometimes correlate across the board with criterion measures is that both types of measure are evaluative. Put differently, the fact that the factor scales of the NB5I were more specifically related to Core Self-Evaluation and Life satisfaction suggest that evaluatively neutralized personality measures such as the NB5I have higher discriminant validity, due to lower intercorrelation between subscales. This is good news for those concerned with revealing theoretically valid relationships between personality variables and relevant criteria, which can be problematic particularly in cases where the criteria are evaluatively loaded, e.g., when it is based on the respondent’s own ratings (Ludeke et al., 2014, for a different but related approach, in the political domain). When an evaluative self-report inventory is related to evaluatively loaded criteria, correlations will be inflated and interpretation in terms of personality content problematic. However, it should be noted that evaluativeness needs not to be a problem in all contexts. In some applied settings, where the primary goal is not to reveal the correlations that are theoretically valid but to make a good decision, e.g., select the most productive applicant, it may actually be useful to have evaluative predictor measures, which, for instance, may capture relevant self-enhancement tendencies.

One notable limitation with these results is that the analyses were only based on two different evaluatively neutralized Big Five inventories that were compared against a small sample of standard inventories, and that the comparison was only made in two different domains of evaluative individual differences (Subjective Well Being and Core self-evaluations). There is reason to expand the current approach to a more systematic study of how evaluatively and neutralized inventories perform in relation to criteria from various evaluative domains. Although it is difficult to provide an estimate of how large the problem of overestimating correlations due to evaluativeness is, clearly a very large part of the research publications in personality- and social psychology contain self-ratings (Baumeister et al., 2007).

Another issue for future research concerns the content of the evaluative factor. Although the current research found support for the evaluative factor and for some of the consequences that is has for discriminant validity, it has less to say regarding what the factor consists in. This is a central question in personality psychology which has caused much debate, e.g., in the literature on the GFP. We join those who argue that the evaluative component is the most crucial. Similarly, Leising et al. (2015) have suggested that the evaluative factor can be seen as a liking factor; those who tend to show that they like themselves or others use the evaluative content of items to express this. But although Leising et al.’s take on the evaluative factor is very similar to what we suggest here, there is one important difference; they suggest that all person judgments are a mixture of content and evaluation. The evaluative factor proposed here is a separate factor, which although somewhat correlated with the core self-evaluative factor is based on the general evaluative content in many items from different personality factors. The core self-evaluative factor has content that is separate from the evaluative factor and should be intact after controlling for the evaluative factor. In this way our results are in line with what Leising et al. have suggested. A minor difference could be that we propose that some people use this evaluative information in items, perhaps unintentionally, to appear as someone who lives up to the societal norm. This is not necessarily the same as describing oneself as someone who likes oneself or someone who has high self-esteem. It is more akin to impression management and self-deception, i.e., socially desirable responding (Paulhus, 1984), although not necessarily simply a response style, but more like self-enhancement (Kwan et al., 2004).

## Discussion

The present research concerned the consequences of social desirability in five factor scales, and what can be done about it. Our strategy was to reduce the evaluativeness of the items, and we showed that more neutral scales have advantages such as items with reduced desirability (Study 1), lack of higher order factors (Study 2), and higher discriminant validity in relation to concept measured with obviously evaluative instruments (Study 3, e.g., Core self-evaluation).

### The Utility of Evaluatively Neutralized Inventories

In research that concerns the role of different aspects of personality for a given phenomenon, such as in Study 3 on core self-evaluations, pure measurement is essential. In such cases, validity hinges on the ability to separate different sources of variation represented in the theoretical model and excluding variance that is not part of the model (such as social desirability). Personality and evaluation should be treated as separate constructs and measured separately for psychometric reasons. Put differently, the FFM should be estimated free of evaluation since it makes the factor scales less correlated. Many personality inventories are susceptible to the influence of socially desirable responding, and the responses constitute a mix between description and evaluation. This put limits on the conclusions that can be drawn from the research where they are used. Separating variation related to individual differences in personality content from individual differences in factors related to evaluation is thus not only a psychometrical issue but an issue for all who are in the business of testing personality theory.

In addition to the standard issues of test validation, there is a separate issue when it comes to evaluative neutralization – it is a limitation of the neutralization method that there is no guarantee that a neutralized item will remain neutral when translated verbatim. The valence-related connotations of the item may very well differ between cultures, not least since there are cultural variations in social norms and thereby variations in which personality traits that are considered desirable. Variations in social norms are likely to affect item popularity, which, as the current results support, is an indicator of the item’s social desirability. Item popularity is a useful criterion when rephrasing the item to make it more neutral (Bäckström and Björklund, 2013) and for selecting among existing items when the mean rating level of the population is known. Creating evaluative neutralized five-factor inventories in French, German, Chinese and other major languages will require calibration of the items, and we suggest aiming for items with mean ratings that are close to the midpoint of the rating scale in the given culture. Fortunately, the neutralization method is fairly simple to apply, as indicated by the finding that when psychometrically naïve undergraduate students were asked to rephrase personality items, the correlation between the big five factors decreased, as did the relations to social desirability (Bäckström and Björklund, 2013). Also, let’s not forget about the alternatives to evaluative neutralization. Some personality measures, such as Jackson’s PRF (Jackson, 1984) and the HEXACO (de Vries, 2011), are relatively free from socially desirable responding. Furthermore, better instruments may be created using items and scales already available that are relatively neutral.

We are not suggesting that evaluative five factor inventories always result in biased predictions. Rather, we want to stress the importance of making a conscious decision when selecting among the many five-factor instruments that exist today. All of them capture the same basic model, but they differ from one another. The strengths and weaknesses of the instrument will have consequences on the results of the research. Evaluatively neutralized instruments are useful when the factor structure is central to the research question, and there is a focus on discriminant validity, e.g., when the criteria are evaluative.

As regards applied contexts, these afford different motivations than the typical self-ratings in basic research do. In applied contexts social desirability can arise as a consequence of external affordances (such as in a recruitment situation) and there are different opinions regarding whether the influence of social desirability on the relationship between personality and work-related criteria is harmful or not. For example, Connelly and Chang (2016) found that social desirability, after controlling for personality content, is negatively related to performance criteria. Others have found that social desirability generally shows weak relations to both personality-related (Piedmont et al., 2000; Paunonen and LeBel, 2012) and performance-related criteria (Ones et al., 1996). The issue is not settled yet and additional research is needed. It is a limitation of evaluatively neutralized inventories that it is still an open question whether such instruments are less influenced by contextually induced social desirability. Further research will tell which methods that are most useful for reducing faking and socially desirable responding in which of the many contexts where personality is being measured.

Arguably, if a scale constructor is interested in qualities related to evaluativeness, this variability could be measured separately, in addition to neutralized personality measures. A possible objection to this, however, is that neutralization comes with a cost – namely that it decreases the room for extreme scores on the negative side of the response scale or makes the scales less reliable on some part of the trait dimension (Wood and Furr, 2016). This critique rests on the argument that neutralization decreases variability, which does not appear to be the case (Bäckström et al., 2014). Instead, the use of evaluatively neutralized items, where mean ratings are closer to the midpoint of the response scale in the general population, seems to optimize variability on both ends of the factor, e.g., both introversion and extraversion. In the Bäckström et al. (2014) study, the criterion validity of the neutralized inventory, as captured by means of an extensive set of criterion measures (from Botwin and Buss, 1989;, Goldberg 2010 and Paunonen, 2003), was found to be very similar to that of the original inventory. Thus, at present the contention that evaluative neutralization throws out the baby with the bathwater is not supported by the empirical results.

More generally, we should not forget that personality inventories rely on self-ratings and that attempts to make them completely resistant to socially desirable responding or lying is a utopian scheme. Although evaluative neutralization makes it difficult for the rater to identify which response to a given item is the most socially desirable, it is still possible to engage in strategic self-presentation.

## Conclusion

Throughout the history of personality assessment, self-rating inventories have been criticized for being subjective to the influence of social desirability. An important cause of socially desirable responding is that popular items will trigger social desirability concerns for some people and result in scales where content and evaluation are mixed, which compromises the measurement of the trait-related contents of the included scales (Bäckström and Björklund, 2013). Using evaluatively neutralized inventories, where the items have been phrased in a less evaluative manner than is usually the case, is one way of trying to contend with the influence of socially desirable responding. The neutralized big five instruments (NB5I) capture personality content equally as well as other five-factor tests do but with less social desirability. We hope that such inventories can contribute to the field by allowing purer measurement and thereby more valid personality data. The use of self-ratings is likely to continue to be a main means of capturing people’s feelings, thoughts and behavior in future personality research, which is why it is so important to keep attempting to improve the method.

## Data Availability Statement

The datasets presented in this study can be found in online repositories. The names of the repository/repositories and accession number(s) can be found below: https://www.openicpsr.org/openicpsr/project/106360/version/V6/view.

## Ethics Statement

The studies involving human participants were reviewed and approved by Swedish Ethical Review Authority. The patients/participants provided their written informed consent to participate in this study.

## Author Contributions

MB and FB contributed to study conceptualization, data collection, and report writing. MB made the main part of data analyses and preparation. Both authors contributed to the article and approved the submitted version.

## Conflict of Interest

The authors declare that the research was conducted in the absence of any commercial or financial relationships that could be construed as a potential conflict of interest.

## Footnotes

1 Data from all studies can be downloaded from https://www.openicpsr.org/openicpsr/project/106360/version/V6/view.

2 Due to a failure in the application used 6 items of from the evaluative inventory and 4 items from the neutralized inventory was not recorded in the database. The lost items were distributed among all scales.

3 We created different versions of parcels based on random allocation, which all generated the same pattern of results.

4 We also performed the analysis with the careless responders included, with very similar results.
